# Challenges in Conducting Quantitative Patient-Centered Benefit-Risk Assessments: A Case Study in Ph + ALL with Immature Efficacy Data

**DOI:** 10.1007/s43441-026-00935-x

**Published:** 2026-03-09

**Authors:** Ajibade Ashaye, Caitlin Thomas, Vamsi Kota, Nicolas Krucien, Kevin Marsh

**Affiliations:** 1 Takeda Development Center Americas, Inc., 500 Kendall Street, Cambridge, MA 02142 USA; 2 PPD Evidera Patient-Centered Research, Thermo Fisher Scientific, London, UK; 3 Section of Hematology & Oncology, Georgia Cancer Center at Augusta University, Augusta, GA USA

**Keywords:** Benefit-risk assessment, Discrete choice experiment, Leukemia, lymphoblastic, acute, philadelphia-positive, Patient preference, Patient-centered outcomes research

## Abstract

Quantitative benefit-risk assessment (qBRA) can reveal how patients balance benefits and risks of cancer treatments. To align with qBRA good practice guidelines, researchers must address challenges including attribute value dependence, double counting, attribute dominance, and uncertainty associated with immature clinical trial outcomes. We present a case study illustrating these challenges in a qBRA of treatment preferences among patients with Philadelphia chromosome-positive acute lymphoblastic leukemia. Preferences were elicited using a discrete choice experiment (DCE). First, we explain how we mitigated potential dominance of survival outcomes by narrowing the range of overall survival (OS) durations that each participant considered. Second, we describe how we acknowledged the conceptual interaction between OS and duration of remission (DOR) attributes and tested for a statistical interaction. Third, we detail how we conducted qBRA with uncertain efficacy data using bivariate sensitivity analysis. Bivariate sensitivity analysis based on DCE-elicited preferences and head-to-head clinical performance data showed that if the considered treatments – ponatinib + chemotherapy and imatinib + chemotherapy – had equivalent efficacy, 52.9% (95% CI: 52.5%–53.4%) of DCE participants would be expected to choose ponatinib over imatinib. If ponatinib offered 10-month longer DOR and 20-month longer OS vs. imatinib, 71.6% (95% CI: 67.2%–76.0%) would choose ponatinib. Probabilistic sensitivity analyses showed that the probability of  ≥ 70% of patients preferring ponatinib is 77.5% if ponatinib offers 15-month longer OS and DOR and 93.0% if it offers 45-month longer OS and DOR. Preference heterogeneity analyses identified that the overall choice probability results hold for all subgroups in nearly all scenarios.

**Supplementary Information:**

The online version contains supplementary material available at 10.1007/s43441-026-00935-x.

## Introduction

Quantitative benefit-risk assessment (qBRA) can provide insights into how patients balance the benefits and risks of a medical treatment [1, 2]. To conduct qBRA, patients’ preferences for benefits and risks of the treatment must first be elicited with a preference elicitation technique such as a discrete choice experiment (DCE), swing weighting, or thresholding [2]. Preference weights for benefits and risks are then calculated by combining the results of the elicitation exercise with data on treatment efficacy and safety to generate patient-centered, decision-relevant behavioral insights, such as net benefit-risk or predicted choice probabilities (PCPs) [1, 2].

Compared to prior efforts to consider trade-offs between survival and factors that may impact quality of life, patient-centered qBRA provides a systematic and transparent approach to integrate elicited patient preferences and clinical evidence, to estimate the net benefit-risk score of a treatment or predicted treatment choices. This approach aligns with International Council for Harmonisation (ICH) and US Food and Drug Administration efforts supporting the incorporation of patient preferences into benefit-risk evaluations [3–5] and provides insights from a larger pool of patients than that typically involved in providing the patient perspective in regulatory or health technology assessment decision-making. Evidence from patient-centered qBRA can be used to better understand patients’ priorities and thereby support regulatory decision-making on a treatment’s efficacy and safety [2, 4, 6–10], align drug development and clinical trial design with patients’ priorities, and inform discussions with patients. To support the use of qBRA, an International Society for Health Economics and Outcomes Research (ISPOR) Task Force recently published guidance highlighting good practices in the design, conduct, and reporting of qBRA [2]. To promote and assess compliance with good practices, publications of qBRA should discuss the process of methods development and the rationale for methods selected [2].

Here, we aim to outline challenges in conducting qBRA and to illustrate how these challenges can be overcome by following qBRA good practices [2]. We focus on a key feature of qBRA good practice: qBRA design must be based on a good conceptual understanding of patients’ preferences and an appreciation of the clinical data that the qBRA is designed to interpret. We center our discussion on a case study detailing the design of a qBRA in Philadelphia chromosome-positive acute lymphoblastic leukemia (Ph + ALL) in which preferences were elicited using a DCE [11]. qBRAs can face common challenges, in particular, avoiding value dependence and double counting and dealing with the uncertainties associated with immature clinical trial data [2]. In this article, we define these challenges further, explain how they can confound the conduct of reliable qBRA, and illustrate and test approaches for addressing them. In doing so, we hope to encourage the adoption of good practice when conducting qBRA, with a special focus on conducting qBRA in oncology when efficacy data are immature.

## Case Study in Designing a qBRA in Oncology

### Overview of Case Application

Tyrosine kinase inhibitor (TKI)-based treatments have reshaped the treatment landscape for patients with Ph + ALL, dramatically improving survival and disease-free survival in patients who had historically faced a dismal prognosis [12]. When patients and their doctors select a frontline therapy for Ph + ALL, they face a decision between several available TKI-based treatments that offer different benefit-risk profiles [13–18]. The Phase 3 PhALLCON trial (NCT03589326) [14] is currently the only trial that has compared TKI treatments head-to-head in patients with newly diagnosed Ph + ALL. PhALLCON was conducted to assess the efficacy and safety of ponatinib + reduced-intensity chemotherapy vs. imatinib + reduced-intensity chemotherapy (henceforth, “chemotherapy”) [14], 2 treatments that had been used off-label as frontline therapy in Ph + ALL [19]. The primary endpoint of PhALLCON, minimal residual disease-negative complete remission (MRD–ve CR) at the end of induction, was reached by a significantly greater proportion of ponatinib-treated than imatinib-treated patients [14], which led to the approval of ponatinib for frontline use in the US [16, 20]. Ponatinib is currently the only TKI combination treatment approved for use in newly diagnosed Ph + ALL [16, 20].

It remains unclear how patients trade off between the benefits and risks of available treatments, including those used off-label, and which treatment they would ultimately prefer. For instance, ponatinib has a black box warning on its US Food and Drug Administration label for hepatic and cardiovascular (CV) events [16], but is associated with higher levels of efficacy than other TKIs [13, 14]. The goal of the patient preference study was to understand patients’ treatment preferences for frontline therapy, the relative importance that they place on treatment benefits and risks, and the benefit-risk trade-offs they are willing to make when selecting a treatment [11]. The goal of the qBRA was to understand which treatment patients would be expected to prefer based on their elicited preferences [11] and head-to-head clinical data for 2 available treatments [14]. We conducted a qBRA of frontline treatment with ponatinib + chemotherapy vs. imatinib + chemotherapy because these TKI-based treatments are actively used in clinical practice and because they are the only TKI-based treatments with head-to-head clinical data on efficacy and safety [14].

qBRA can be conducted to support different stakeholders, including medical professionals, pharmaceutical companies, health technology assessors, and regulatory authorities, incorporating the patient perspective, and to inform different stages of the drug development cycle [2, 6, 21, 22]. It is critical that the preference study informing the qBRA be centered around the targeted stakeholders and decision-making context [2]. This case study was designed to support medical professionals in shared decision-making and to guide pharmaceutical companies in future treatment development decisions, rather than to support regulatory submissions.

Treatment preferences were elicited in an online DCE [11], conducted in alignment with ISPOR good practice guidelines for preference elicitation research [23, 24] (Online Resource 1). In total, 201 people with Ph + ALL completed the DCE; participants had a mean age of 45 ± 13 years, 60% were male, and 67% were in remission [11].

Briefly, US-based adults with Ph + ALL were recruited for the DCE via physician referrals, databases, social media, and through the Leukemia and Lymphoma Society [11]. The treatment attributes and performance levels used to describe patient-relevant benefit-risk trade-offs were identified through a targeted literature review, interviews with 2 hematologists who treat patients with Ph + ALL, feedback from the Leukemia and Lymphoma Society, including a lay patient representative, and 5 pre-testing interviews with patients [11]. The following attributes were selected: 1) overall survival (OS); 2) duration of remission (DOR); 3) risk of a major CV event (i.e., myocardial infarction or stroke); and 4) risk of myelosuppression (Table 1) [11].

**Table 1:** Attributes, definitions, and levels included in the DCE

Attribute (patient-friendly wording)	Definition (abbreviated)	Levels
Overall survival	Number of months that participants can expect to stay alive after starting treatment	Variant A: 30, 35, 40, 45, 50, 55, 60 Variant B: 60, 65, 70, 75, 80, 85, 90
Duration of remission	Number of months that participants can expect to stay in remission after achieving remission	Variant A: 15, 20, 25, 30, 35, 40, 45 Variant B: 45, 50, 55, 60, 65, 70, 75
Risk of a major cardiovascular event	Likelihood of experiencing a major cardiac event, in particular heart attack and stroke. This risk applies during the first 6 months of treatment	Both variants: 0%, 25%, 50%
Risk of myelosuppression (suppressed immune system)	Likelihood of experiencing a decrease in bone marrow activity that could result in fatigue or an increased risk of infection. Myelosuppression results in: feeling tired, feeling short of breath, getting infections more easily (potentially requiring hospitalization), experiencing fever, increased bruising, and bleeding more easily. This risk applies during the first 6 months of treatment	Both variants: 0%, 50%, 100%

DCE, discrete choice experiment

Operationalizing the designs of our DCE and qBRA in compliance with qBRA good practices [2] posed several challenges. Below we discuss 3 key challenges.

### Challenge 1: Dominance of Survival Outcomes

To elicit preference weights for attributes within a qBRA, elicitation exercises need to stimulate trade-off behavior by ensuring that single attributes do not dominate decision-making [2, 25, 26]. If choices are dominated by 1 attribute, participants may ignore other attributes and therefore not make trade-offs. When eliciting preferences for cancer treatments, large changes in efficacy (e.g., OS) may cause participants to always prioritize that attribute.

Within the case study, OS levels ranged from 30 to 90 months and were informed by clinical trial data for TKIs and input from a key opinion leader. Adopting a common practice in DCE design and identifying 3 performance levels to cover this range would have resulted in participants being presented with treatment profiles that differed by ≥ 30 months of OS. Given the importance of OS in oncology [27], there is increased risk that OS might dominate if participants are asked to choose between alternatives with widely different levels of OS. Thus, we constrained the difference in OS presented in any choice task in 2 ways. First, we ran 2 variants of the DCE to spread the performance range across participants. Participants completing variant A were shown OS levels ranging from 30–60 months; participants completing variant B were shown levels ranging from 60–90 months. This limited the maximum difference seen across treatment alternatives to 30 months rather than 60 months and reduced any anchoring bias. Second, we limited the maximum OS difference between the pair of treatment alternatives in a task to 10 months, thus increasing the likelihood that other attributes would be considered.

Pilot interviews were conducted to ensure that this approach was effective. In the final DCE, only 4% (n = 8) of participants’ decision-making was dominated by 1 attribute; 5 participants always prioritized DOR, and 3 always prioritized OS. Analyses confirmed that treatment preferences did not significantly differ between the 30–60 months and 60–90 months DCE variants and thus, that the 2 variants could be combined (Online Resource 1).

### Challenge 2: Preferentially Dependent and Overlapping Benefit Outcomes

Preference studies with attributes that are preferentially independent and nonoverlapping have greater statistical and response efficiency [2]. Where attributes do not display these properties, a recommended first step is to redefine the attributes to ensure compliance [2]. However, this is not always possible, in which case the study would need to be designed such that the interaction between attributes could be estimated [2].

**Fig. 1:**
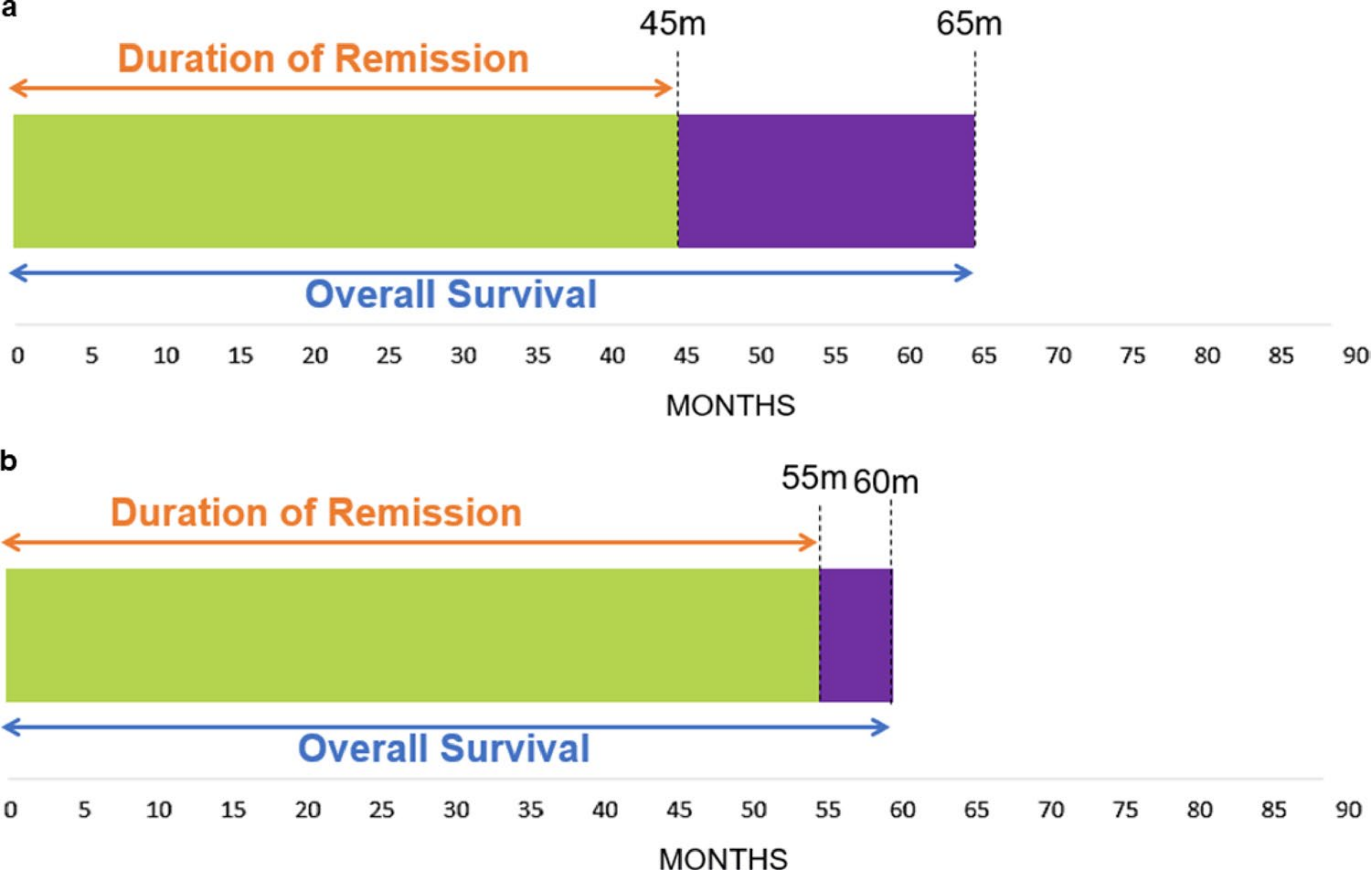
Example timelines presented in the DCE showing overlap between DOR and OS. Example timelines presented in the DCE to show the overlap between DOR and OS **a** with 65 months of OS, of which 45 months are spent in remission, and **b** with 60 months of OS, of which 55 months are spent in remission. Abbreviations: DCE, discrete choice experiment; DOR, duration of remission; OS, overall survival

Preference studies are often subject to preferentially dependent and overlapping attributes. In oncology, the value that participants place on improvements in OS may depend on whether and to what extent this added survival time is spent in remission. As such, changes in OS and DOR attributes may be preferentially dependent. Additionally, OS and DOR overlap, as time in remission is part of OS; this presents a risk of double counting. To estimate any interaction (i.e., second-order) effects between OS and DOR – whether being in remission impacts the value attached to improvements in OS – and avoid double counting the values of OS and DOR, we first needed to ensure that participants understood the conceptual relationship between these attributes. To this end, participants were shown DOR and OS in a timeline that depicted how DOR was part of OS (Fig. 1) [11]. In addition, we specified plausibility constraints in our experimental design to improve the realism of the choice tasks. For instance, our design ensured that OS was always longer than DOR. Testing during pilot interviews showed that patients understood the relationship between DOR and OS.

We expected that sensitivity to marginal improvements in OS would increase with longer DOR. However, no significant interaction effect was detected (*p* = 0.80), suggesting that participants’ valuation of increases in OS was independent of whether an increase in OS was accompanied by an increase in DOR.

### Challenge 3: Immature Efficacy Data

qBRA integrates performance data and preference estimates to generate an overall patient-centered assessment of the benefit-risk balance of a treatment [2]. Where performance outcomes such as OS are immature, as is often the case in oncology [28], it is not possible to generate an overall assessment through standard base case analyses [2]. Alternative analyses are therefore needed to explore how outcome uncertainty impacts patients’ assessment of the benefit-risk balance [2].

In our case application, median OS data were immature for both treatment arms, and median DOR data were immature for the ponatinib + chemotherapy treatment arm (Table 2) [14]. Various approaches are available to estimate the impact of uncertainty. One option would be to reflect this uncertainty in DCE attributes and levels – for instance, defining a level of OS as above a certain level, but uncertain. This would formally elicit patients’ preferences for such uncertainty. However, we adopted an alternative strategy because uncertainty in efficacy is expected to resolve when more clinical data become available. Lower ends of efficacy ranges were informed by trial data, but because medians had not been reached, the upper ends of efficacy ranges were informed by input from key opinion leaders to cover the likely outcome ranges. Different sensitivity analyses were conducted to understand how alternative levels of OS and DOR would impact the benefit-risk balance. Each type of sensitivity analysis provided complementary insights into the predicted uptake of ponatinib relative to imatinib. The deterministic bivariate sensitivity analysis allowed us to directly manipulate the levels of additional OS and DOR to quantify predicted uptake under fixed combinations of these parameters. By showing which combinations of OS and DOR were associated with the same level of predicted uptake, this analysis revealed how participants trade off between gains in OS and DOR. In contrast, the probabilistic sensitivity analysis incorporated uncertainty in the levels of additional OS and DOR by modeling OS and DOR as distributions rather than fixed values. This approach allowed us to estimate predicted uptake under scenarios reflecting “small”, “medium”, and “large” gains in efficacy with ponatinib over imatinib. These sensitivity analyses were used to progressively build a more comprehensive picture of decision-making under different possible conditions. This structured, stepwise approach aligns with the framework described in the ISPOR good practices report for qBRA, in which analyses evolve from simple to more complex [2].

**Table 2:** Performance matrix comparing ponatinib + chemotherapy vs. imatinib + chemotherapy

Treatment profile	Median OS in months (95% CI) [14]	Median DOR in months (95% CI) [14]	Major CV event risk^a^ (%) [14]	Myelosuppression risk^b^ (%)
Ponatinib + chemotherapy	NR (NR, NR)	NR (NR, NR)	1.84	40.5
Imatinib + chemotherapy	NR (29.04, NR)	22.32 (19.04, NR)	1.2	54.3

CI, confidence interval; CV, cardiovascular; DOR, duration of remission; NR, not reached; OS, overall survival

^a^Treatment-emergent cardiovascular or cerebrovascular arterial occlusive event

^b^Takeda data on file for treatment-related myelosuppression in the PhALLCON trial

First, we ran a deterministic bivariate sensitivity analysis using preference data from the DCE (Online Resource 1; Online Resource 1: Table S1) [11], safety data from the PhALLCON trial [14], and potential ranges for improvements in DOR and OS offered with ponatinib + chemotherapy over imatinib + chemotherapy. Longer DOR with ponatinib was supported by PhALLCON data showing that median DOR was reached for imatinib, but not ponatinib [14]. Longer OS with ponatinib was supported by significantly longer OS in an matching-adjusted indirect treatment comparison [13] and by a higher proportion of ponatinib- than imatinib-treated patients in PhALLCON achieving MRD–ve CR [14], which is strongly correlated with long-term OS [29]. In total, 427 scenarios were run, reflecting 1-month increment increases in OS provided by ponatinib over imatinib over a range of 0–60 months and 10-month increment increases in DOR over a range of 0–60 months.

**Fig. 2:**
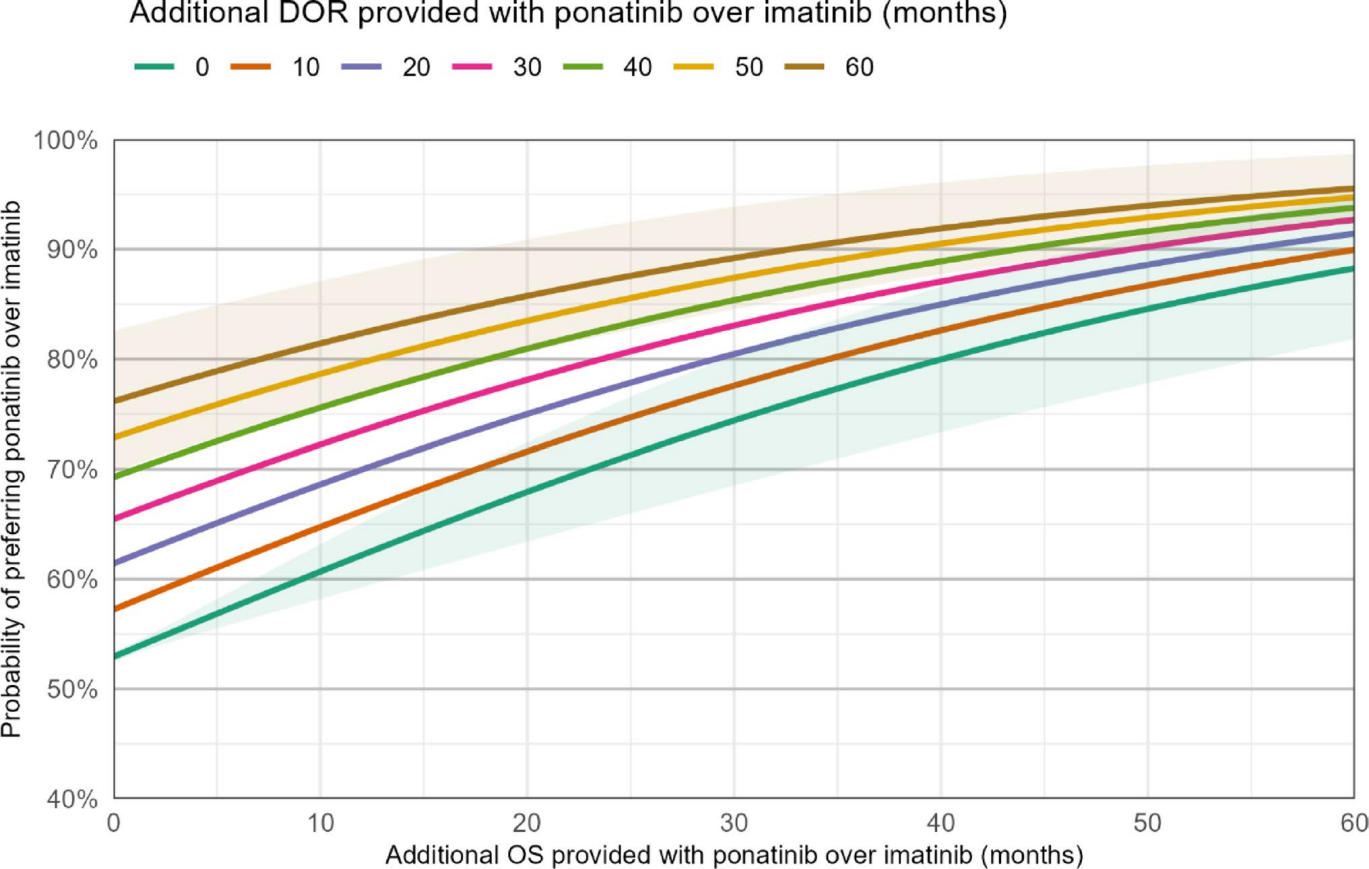
A bivariate sensitivity analysis of the probability that participants would prefer ponatinib + chemotherapy over imatinib + chemotherapy. Predicted probabilities of preferring ponatinib + chemotherapy over imatinib + chemotherapy under different potential levels of additional OS and DOR provided with ponatinib over imatinib. Shading indicates 95% confidence intervals for 0 (green) and 60 (brown) additional months of DOR provided with ponatinib over imatinib. Abbreviations: DOR, duration of remission; OS, overall survival

The bivariate sensitivity analysis indicated that if ponatinib and imatinib had equivalent efficacy, 52.9% (95% confidence interval, 52.5%–53.4%) of study participants would be expected to select ponatinib over imatinib, and that the proportion preferring ponatinib would increase substantially with greater gains in OS and DOR with ponatinib relative to imatinib (Fig. 2). The modest preference for ponatinib over imatinib in the equivalent efficacy scenario was driven by the 13.8% lower risk of myelosuppression with ponatinib than with imatinib, which outweighed the 0.6% higher risk of major CV events with ponatinib (Table 2) [14]. Critically, the sensitivity analysis allowed us to explore how the likelihood of preferring ponatinib increases for different combinations of improvements in OS and/or DOR. For instance, if ponatinib offers 10 months of additional DOR *and* 20 months of additional OS, the likelihood of ponatinib being preferred would increase to 71.6% (95% confidence interval, 67.2%–76.0%).

**Fig. 3:**
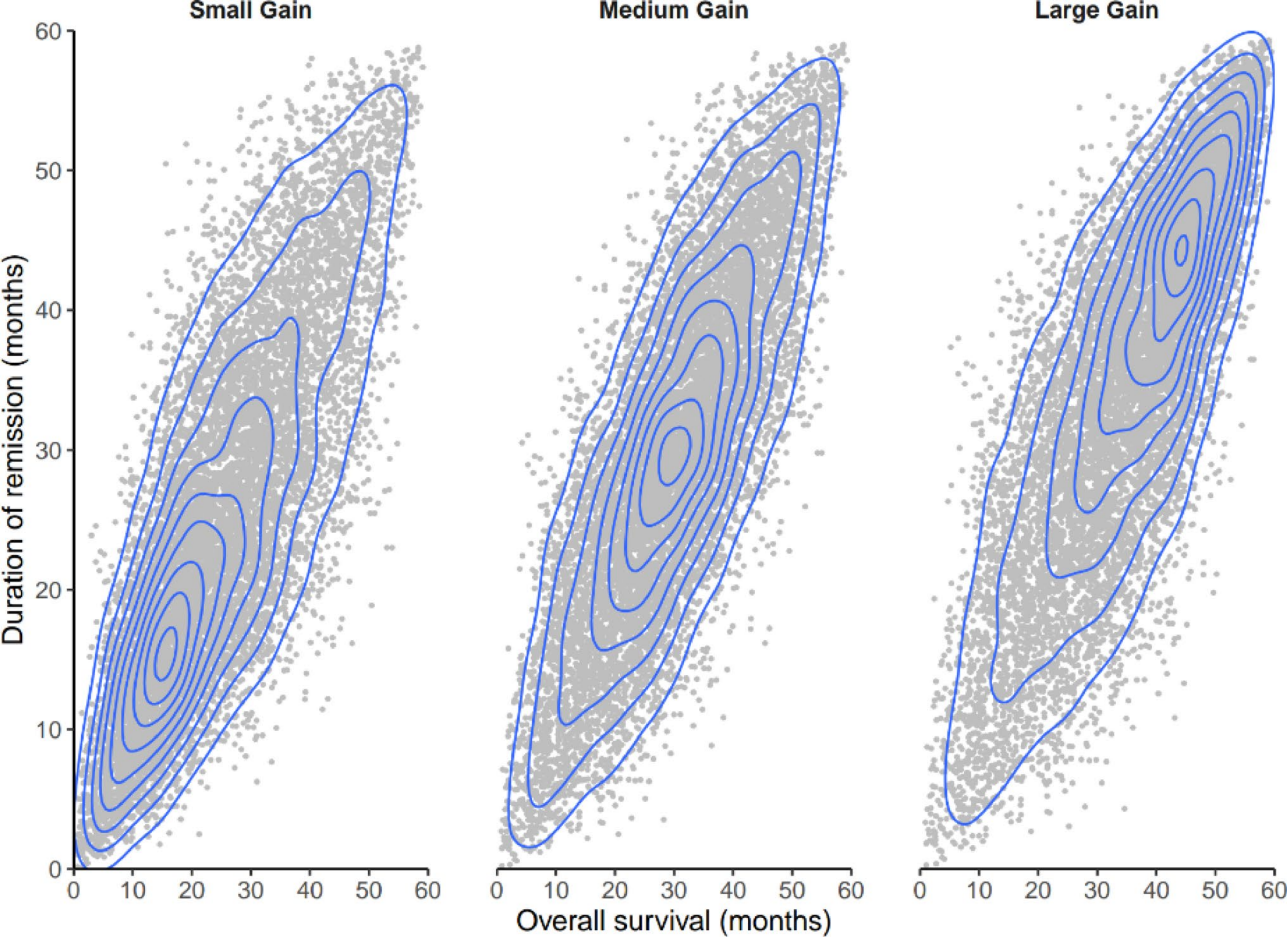
Probabilistic sensitivity analysis: Distributions of simulated performance under different assumptions of additional OS and DOR. Distributions of simulated additional OS and additional DOR provided by ponatinib over imatinib in 10 000 simulated cases under small gain, medium gain, and large gain scenarios. The small gain scenario assumes that the values of additional OS and DOR provided by ponatinib over imatinib follow a triangular distribution peaking at 15 months. The medium gain scenario assumes a triangular distribution peaking at 30 months, and the large gain scenario assumes a triangular distribution peaking at 45 months. No constraints were imposed on the relationship between OS and DOR, except that the 2 triangular distributions were defined to be highly correlated (correlation = 0.8). Abbreviations: DOR, duration of remission; OS, overall survival

**Fig. 4:**
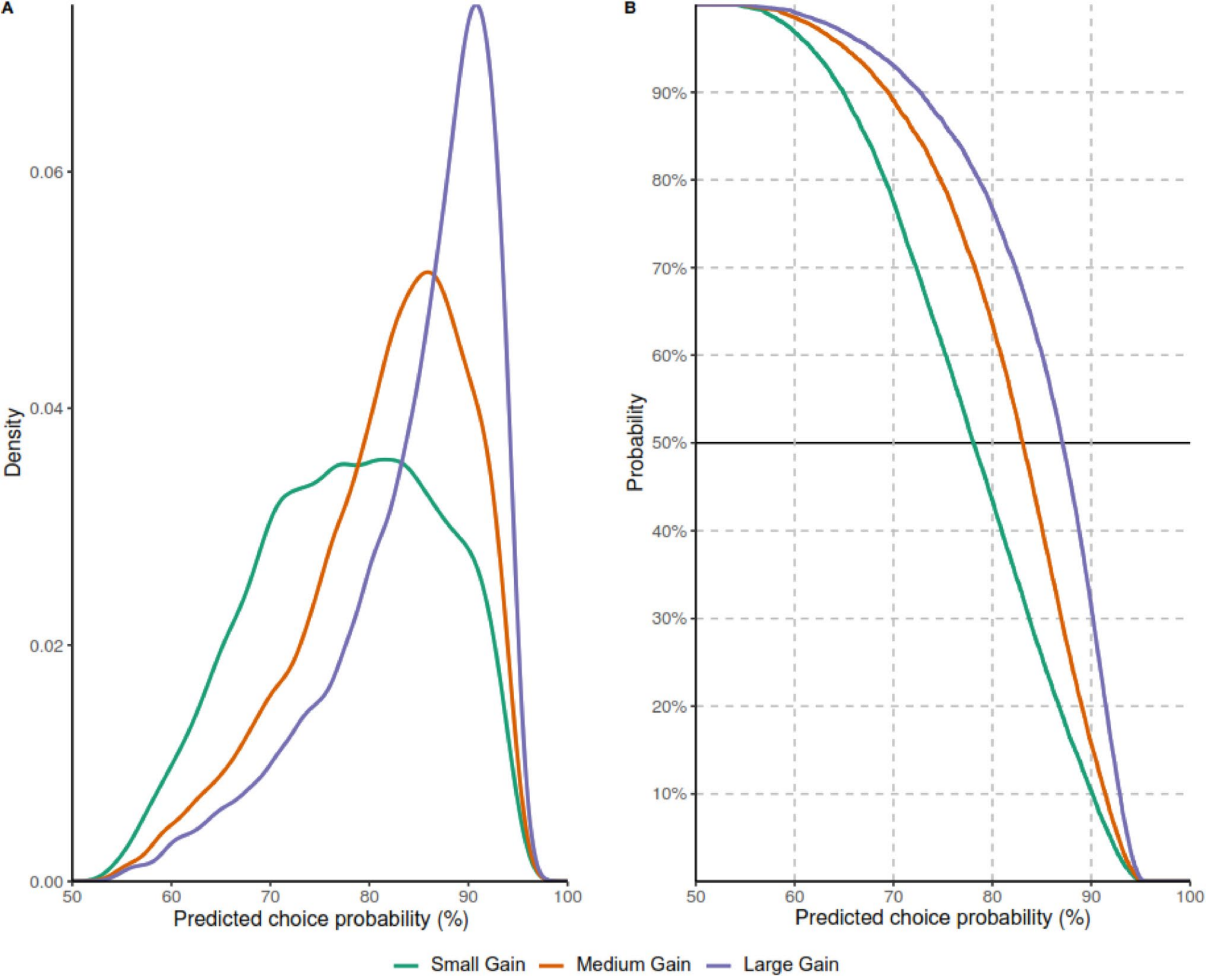
Probabilistic sensitivity analysis: Probability density and cumulative distribution functions of predicted choice probabilities for ponatinib. **A** Probability density function and **B** complementary cumulative distribution function of predicted probabilities for choosing ponatinib over imatinib under scenarios where ponatinib offers a small gain, medium gain, or large gain in efficacy over imatinib. The small gain scenario assumes that the values of additional OS and DOR provided by ponatinib over imatinib follow a triangular distribution peaking at 15 months. The medium gain scenario assumes a triangular distribution peaking at 30 months, and the large gain scenario assumes a triangular distribution peaking at 45 months. No constraints were imposed on the relationship between OS and DOR, except that the 2 triangular distributions were defined to be highly correlated (correlation = 0.8). Abbreviations: DOR, duration of remission; OS, overall survival

We conducted probabilistic sensitivity analyses to explore how variation in efficacy parameters impacted PCPs (Fig. 3–4). To reflect potential differences in the additional OS and DOR provided by ponatinib over imatinib, we drew 10 000 values from correlated triangular distributions of potential additional OS and DOR provided by ponatinib over imatinib, with a minimum difference value of 0 and a maximum difference value of 60 (Online Resource 1). The use of triangular distributions was selected as most appropriate because it ensured that ponatinib could not have worse OS or DOR than imatinib (as was expected based on data from the PhALLCON trial [14, 29]) and that values could not exceed the maximum possible differences in OS and DOR defined in the DCE (Online Resource 1).

At each iteration, we computed the probability of preferring ponatinib over imatinib. The choice probability for ponatinib, accounting for uncertainty in the additional OS and DOR provided by ponatinib over imatinib, was computed as the average over the 10 000 PCPs.

This analysis was performed under 3 scenarios, where each scenario corresponded to different modes of the triangular distributions (Fig. 3). The values selected as the peaks of the triangular distributions (i.e., 15, 30, and 45 months) were chosen to cover the 0 to 60 month range of differences in OS and DOR values included in the DCE. The “small gain” scenario assumed that the additional gain offered by ponatinib followed a triangular distribution with a peak at 15 months of additional OS and DOR over imatinib (i.e., 15 months was the most common additional gain with ponatinib) (Online Resource 1). The “medium gain” scenario was in between the extreme scenarios, with a triangular distribution peaking at 30 months. The “large gain” scenario assumed that the triangular distribution peaked at 45 months.

These scenarios resulted in different distributions of PCPs (Fig. 4A). The average (standard deviation) PCP for ponatinib was 77.7% (9.3) in the small gain scenario, 81.6% (8.4) in the medium gain scenario, and 84.7% (8.1) in the large gain scenario. These distributions can be used to determine the probability of achieving a minimum level of uptake for a treatment (here, ponatinib). For example, the probability of at least 70% of patients choosing ponatinib over imatinib is 77.5% in the small gain scenario, 89.1% in the medium gain scenario, and 93.0% in the large gain scenario (Fig. 4B).

**Fig. 5:**
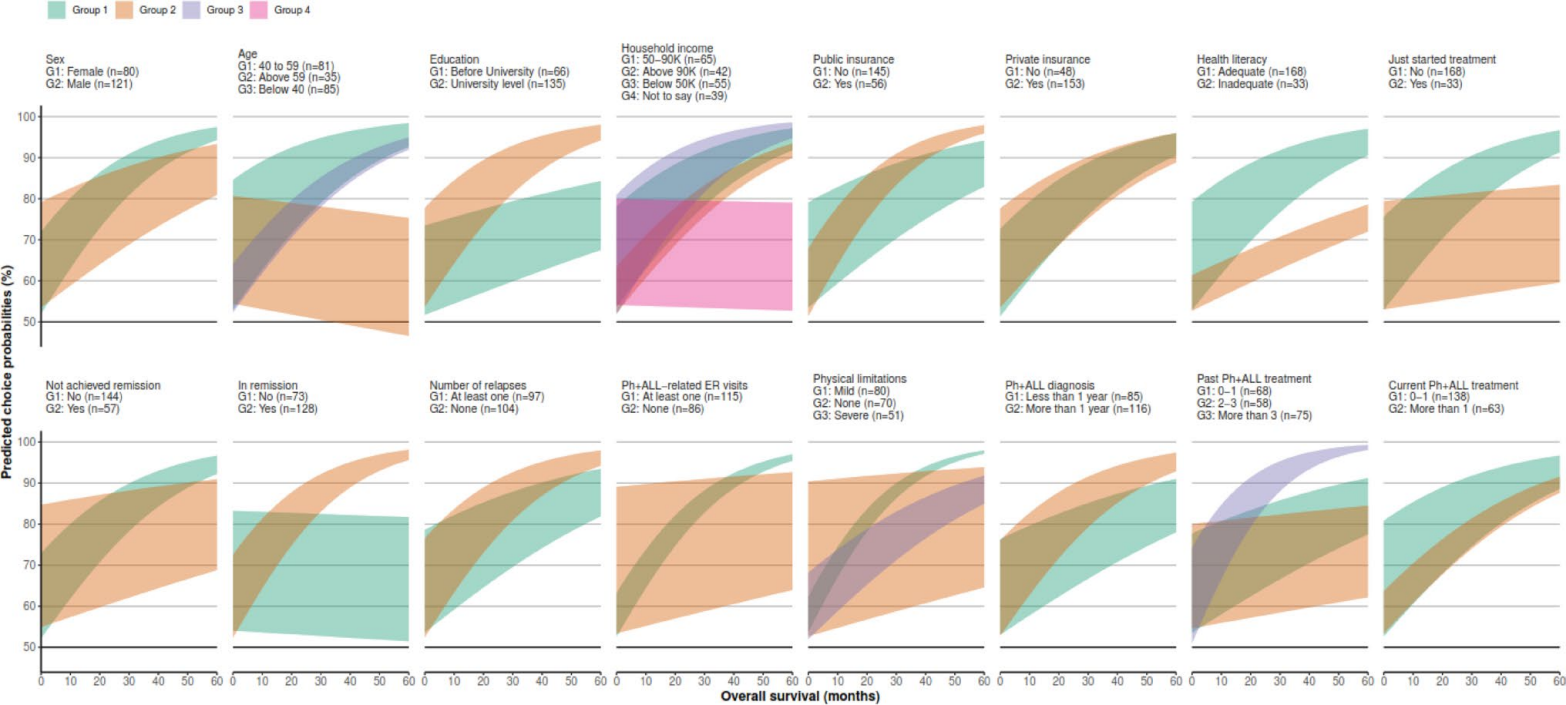
Subgroup analyses of preference heterogeneity: Probabilities that patients with different demographic and clinical characteristics would prefer ponatinib over imatinib. Predicted choice probabilities of preferring ponatinib + chemotherapy over imatinib + chemotherapy. Probabilities are estimated for different potential levels of additional OS and DOR provided with ponatinib over imatinib. Levels of DOR are represented in the height of the colored ribbon, with the bottom of each ribbon representing the minimum additional DOR (0 months) and the top of each ribbon representing the maximum additional DOR (60 months). Abbreviations: DOR, duration of remission; ER, emergency room; G, group; OS, overall survival; Ph + ALL, Philadelphia chromosome-positive acute lymphoblastic leukemia

We explored the impact of preference heterogeneity on PCPs by conducting bivariate sensitivity analyses for subgroups of participants, where subgroups were split based on individual demographic or clinical characteristics (Fig. 5) [2]. These analyses were intended to account for uncertainty in attribute valuation due to the heterogeneous preference weights observed in participants with different personal characteristics (Online Resource 1: Table S2) [11]. For each analyzed subgroup, a bivariate sensitivity analysis was fitted to preference estimates for that subgroup [2]. The overall results – that most study participants would prefer ponatinib over imatinib – held for all analyzed subgroups in nearly all scenarios. Patients aged ≥ 59 years, who placed more weight on DOR and avoiding adverse events, had a small, negative, and statistically insignificant preference for improvements in OS. As such, marginally fewer than 50% of patients aged ≥ 59 years would prefer ponatinib in the scenarios where ponatinib, relative to imatinib, offers larger improvements in OS but either small or no additional DOR. However, most patients aged ≥ 59 years would prefer ponatinib in scenarios where the additional OS offered by ponatinib was coupled with larger improvements in DOR, as was the case in the vast majority of scenarios. Although this analysis highlights choice probabilities in different participant subgroups, the relationships between PCPs and personal characteristics are not necessarily causal.

## Discussion

We report on the design of a qBRA for treatment of Ph + ALL to showcase challenges associated with the conduct of qBRAs in oncology and to illustrate how these challenges can be overcome by adhering to good practice guidelines [2]. We explain how common challenges in qBRA – the presence of attribute overlap and preference dependence, the possibility of attribute dominance, and immature clinical study outcomes – can confound the conduct of reliable qBRA, and we describe approaches for addressing each challenge.

Our case study highlights lessons for conducting qBRA. First, it is important to understand conceptually the nature of the preferences being elicited, including the relationship between preferences for treatment attributes and how this relationship corresponds with the assumptions underlying the methods being employed for preference elicitation and analysis. In particular, researchers should consider and test for attribute overlap and preferential dependence. These considerations should influence how choice tasks are presented to patients, the experimental design, and the analysis design. Second, to achieve this understanding of preferences on which to build designs, it is important to work with patients through qualitative research and pilot testing and to have patients and/or patient advocates serve as advisors to studies [30]. In our case study, we incorporated patient input and insights at multiple stages, including through our collaboration with the Leukemia and Lymphoma Society across the project lifecycle [11, 30], cognitive pilot interviews to inform DCE development [11], and qualitative interviews (N = 5) conducted in parallel with the main DCE to help guide our interpretation of the elicited preference data [11]. We acknowledge that the sample sizes for our cognitive pilot interviews and qualitative interviews are modest and encourage researchers to use larger sample sizes when this is feasible or when multiple countries are included. Third, it is important to demonstrate that preference elicitation adheres to the assumptions underlying the design of such exercises. For example, our experimental design and analysis plan tested for attribute dominance and the presence of second-order effects. Finally, uncertainty analysis should address the specific sources of uncertainty in trial data and preference data, including reflecting the relationships between analysis inputs. The use of multiple analyses in a stepwise approach is recommended in qBRA guidance [2] and can help to ensure transparency and robustness.

Some of the strategies that we adopted in our qBRA were successful. First, our strategy to ensure utility balance and prevent OS from strictly dominating other outcomes was successful: only 4% of participants systematically made choices based on only 1 attribute. Second, our sensitivity analyses provided quantitative information on how likely participants were to prefer ponatinib over imatinib. Based on PhALLCON data, both DOR and OS are expected to be longer with ponatinib than imatinib; median DOR was reached for imatinib, but not for ponatinib [14], suggesting longer DOR for ponatinib, and rates of MRD–ve CR were higher with ponatinib than imatinib [14], suggesting longer OS for ponatinib (as this endpoint has been found to be correlated with OS [29]). Given this expected efficacy improvement with ponatinib and that slightly more than half of participants were predicted to prefer ponatinib even if the treatments had equivalent efficacy, we can conclude that more than half of participants would prefer ponatinib over imatinib, even as we await final OS and DOR values. Moreover, the sensitivity analyses show that the predicted share of participants preferring ponatinib would increase substantially with larger gains in efficacy. It cannot be guaranteed that this approach will generate such conclusive guidance in other instances, even with similar levels of uncertainty. Notably, a benefit of our qBRA approach is that when mature data (here, OS and DOR) become available, stakeholders can read off of the existing sensitivity plots to see predicted treatment choices in the context of the most recent clinical data.

The success of our strategies to address potential preference dependence is more ambiguous. We employed various strategies to estimate an expected preferential dependence between DOR and OS but did not detect a significant interaction effect. Interaction effects are difficult to detect statistically in preference studies due to sample size and design constraints. It is therefore unclear whether there truly was no interaction, and our efforts successfully demonstrated this, or whether we were not able to detect such an effect due to limitations inherent to the design of the DCE. Interestingly, subgroup analysis suggested potential preference dependence among participants aged ≥ 59 years; this subgroup considered gains in OS less valuable if they were not accompanied by gains in DOR. Further work could test how reliably interaction effects can be detected in DCEs and how to address potential preference dependence among subgroups.

A further limitation of our case study is that it focused on the conduct of a DCE and related decision analysis. This was the appropriate method in this instance, given the sample size, number of attributes, and the desire to estimate any second-order effects. Different methods may be more appropriate under different circumstances. In particular, in rare disease research, it may not be feasible to identify a sample large enough to conduct a DCE. Other methods (e.g., thresholding, swing weighting) can be adopted to elicit preferences when sample sizes are small. Future studies could explore adherence to good practice when employing these methods. Another limitation of our case study is that our sample does not fully match the targeted patient population. Aligning a DCE study population with a narrowly defined target population is an inherent challenge in preference research, particularly in relatively rare patient populations. It would be unrealistic to recruit a sufficiently large sample of newly diagnosed patients with an aggressive cancer like Ph + ALL, given that treatment typically begins as soon as possible after diagnosis. In our case study, only 8% (n = 16) of our sample was newly diagnosed (i.e., received no prior treatment for Ph + ALL), even as the DCE sought to understand decision-making in newly diagnosed patients. Although participants were instructed to make treatment decisions as if they were newly diagnosed, past treatment experiences may have influenced their choices. However, we found that in all subgroups split by the number of prior treatments they had received, the proportion of participants expected to prefer ponatinib increased as OS increased.

Our case study was designed to support medical professionals and pharmaceutical companies in understanding what treatment patients would prefer and how decisions were driven by patients’ benefit-risk trade-offs and the efficacy and safety of available treatments. Consequently, we selected attributes based primarily on their importance to patients. OS was selected because it had been identified as a critical consideration for patients and because patients may have more difficulty interpreting efficacy based on rates of MRD–ve CR, the primary endpoint in the PhALLCON trial [14]. Even though OS data were immature, final MRD–ve CR data from PhALLCON were available and supported our expectation of better long-term OS with ponatinib over imatinib [14, 29]. If the qBRA had been intended to support a regulatory submission, we would have needed to select the primary endpoint of the trial, MRD–ve CR, as an efficacy attribute.

Several challenges discussed in this manuscript are common across therapeutic areas. For instance, clinical trials in many therapeutic areas involve outcomes that are preferentially dependent and overlapping. On the other hand, the challenge of immature OS data is especially relevant in oncology qBRA, given that OS is important to patients with cancer [27] and that OS data in oncology trials often takes several years to mature [31]. Immature OS data may be even more prevalent in studies of early-stage cancer, where patients generally are expected to live longer, than in studies of more advanced cancer. We hope the lessons we have drawn from our case study will support qBRA research across oncology and other therapeutic areas but acknowledge that alternative approaches may be required to adhere to good practice.

## Conclusions

This article highlights several nuanced challenges in conducting qBRA in oncology and illustrates potential strategies to support the conduct of reliable qBRA in adherence to qBRA guidelines. Adhering to qBRA guidelines requires thinking carefully about the preference study design and the clinical data available and can generate valuable information to inform regulatory decision-making, drug development, and shared decision-making.

## Supplementary Information

Below is the link to the electronic supplementary material.

Supplementary Material 1

## Data Availability

The datasets analyzed during this study will not be made available because no consent was sought from participants to allow sharing of their data with third parties.
