# Supplementary material for: Challenges in Conducting Quantitative Patient-Centered Benefit-Risk Assessments: A Case Study in Ph + ALL with Immature Efficacy Data
Source: Ther Innov Regul Sci. 2026 Mar 9;60(3):617–28. doi: 10.1007/s43441-026-00935-x (PMC13110195; doi:10.1007/s43441-026-00935-x)
Supplement: Supplementary file 1 — Supplementary Material 1 [file 43441_2026_935_MOESM1_ESM.docx]

# Online Resource 1

**Electronic Supplementary Material for “Challenges in Conducting Quantitative Patient-Centered Benefit-Risk Assessments: A Case Study in Ph+ ALL with Immature Efficacy Data”, *Therapeutic Innovation & Regulatory Science***

Ajibade Ashaye^1^; Caitlin Thomas^2^; Vamsi Kota^3^; Nicolas Krucien^2^; Kevin Marsh^2^

^1^ Takeda Development Center Americas, Inc., Cambridge, MA, USA

^2^ Evidera, London, UK

^3^ Section of Hematology & Oncology, Georgia Cancer Center at Augusta University, Augusta, GA, USA

Corresponding author: Ajibade Ashaye, ajibade.ashaye@takeda.com

## Table of Contents

| **Item** | **Page number** |
| --- | --- |
| Avoiding attribute dominance: Details on combining 30–60 month and 60–90 month variants of the discrete choice experiment | 2 |
| Preference data from the DCE | 4 |
| Probabilistic sensitivity analysis: Additional technical details | 6 |
| Table S1. Attribute preferences calculated in the DCE case application | 8 |
| Table S2. Significant preference heterogeneity by personal characteristic and attribute | 10 |
| References | 13 |

## Avoiding attribute dominance: Details on combining 30–60 month and 60–90 month variants of the discrete choice experiment (DCE)

The change in the range of levels for the overall survival (OS) and duration of remission (DOR) attributes between the 2 variants could have impacted both determinants of participants’ treatment preferences and the consistency of participants’ choices (e.g., if one version were more difficult for participants than the other). Consequently, several measures were used to compare results from the 2 variants before pooling the choice data.

First, the proportions of choices for treatment option A vs. treatment option B were compared between the 2 DCE versions in a chi-squared test. Treatment option A was preferred in 49% of cases in DCE Variant A and in 50% of cases in DCE Variant B. The chi-square test did not reach significance (p=0.55), indicating that choice proportions did not significantly differ between the 2 variants.

Second, the relative attribute importance (RAI) scores and marginal rates of substitution (MRS) were computed based on utility estimates from the linearly coded multinomial logistic regression (MNL) models (Table S1) [1]. The largest RAI difference was obtained for the OS attribute (Variant A = 25%; Variant B = 38%; difference = 13 percentage points). Importantly, a two-sided z-test indicated that this difference was not statistically significant (p=0.121). The risk of myelosuppression appeared to be more important than the risk of cardiovascular (CV) events; this was relatively stable across the variants (Variant A: 34% vs. 20%; Variant B: 29% vs. 18%). This relationship was also reflected in the MRS for CV risk with respect to myelosuppression risk (Variant A: 1.19; Variant B: 1.23). OS also appeared to be more important than DOR, but the size of the difference was influenced by the variant, with MRSs of 1.16 in Variant A vs. 2.40 in Variant B.

Third, a heteroscedastic version of the dummy-coded MNL model was estimated that allowed the utility scale, which is inversely related to the variance of errors and hence the consistency of choices, to vary between the 2 DCE versions. The estimated effect (β=0.25 [SE=0.16]) was not significant (p=0.109), suggesting that the utility scale did not differ significantly between the 2 versions.

These results suggested that treatment preferences did not significantly differ between the 2 variants. This was further confirmed by the small loss of statistical performance when combining the variants. The choice data were compared separately in a split-sample approach, and then jointly, to understand which method had the best statistical performance. The log-likelihood was –795 for the model estimated on the choice data from DCE Variant A (for 5 parameters) and –720 for the model estimated on the choice data from DCE Variant B (for 5 parameters). Therefore, the combined log-likelihood when applying the split-sample approach was –‍1515 (for a total of 10 parameters), giving a Bayesian information criteria (BIC) of 3108. The log-likelihood of the model estimated on the pooled sample was –1522 (5 parameters), and the corresponding BIC was 3083. The joint model appeared to outperform the split-sample approach because it was more parsimonious (i.e., used fewer parameters) and had a better BIC value.

Based on these results, we concluded that neither the utility scale nor the determinants of treatment preferences significantly differed between the DCE variants.

## Preference data from the DCE

Elicited preferences for each attribute are summarized in Table S1 [1]. These preferences and the methods used to derive them have been published [1].

DCE procedures

Briefly, each participant completed 12 experimental choice tasks [1]. Participants chose their preferred treatment option between alternatives that differed on levels of OS, DOR, major CV event risk, and myelosuppression risk. Attribute levels were varied across tasks according to the experimental design. In the first part of each task, participants chose between 2 alternatives: chemotherapy + tyrosine kinase inhibitor (TKI) A and chemotherapy + TKI B. After selecting their preferred treatment option, participants completed an “opt-out” question: “If these treatments were offered to you by your doctor, would you 1) take the treatment that you just chose above or 2) take neither of the treatments?”

DCE data quality

Data quality was confirmed in several tests of internal validity [1]. The vast majority (88%) of participants passed the choice dominance test, choosing the treatment option that was superior on all attributes; this exceeded the expected pass rate of 87%. Over three-quarters (78%) of participants answered repeated choice questions consistently, comparable to or higher than the proportions observed in other DCEs [2-4]. Nearly all (96%) participants considered more than one attribute when making their choices (i.e., did not show lexicographic preferences). Finally, nearly all (98%) participants varied their choices between the 2 choice alternatives (i.e., not choosing only TKI A + chemotherapy or TKI B + chemotherapy across all tasks).

DCE analyses

Statistical performance was compared between model specifications that included only the initial preference choice vs. models that combined the initial and opt-out choices (Supplementary Table 1) [1]. Models including only the initial preference choice had better statistical fits, defined as lower BIC values. As such, preferences were estimated considering initial choices but not opt-out choices.

Attribute levels were linearly coded [1]. Linear encoding assumes linearity in risk preferences, such that a 1-unit change in the attribute has a constant effect on participants’ choices and does not depend on the absolute value of the attribute level. Linearity of the attribute preferences was verified by fitting a linear regression; linearity was accepted because each of the regression coefficients was > 0.7 (OS: 0.82, DOR: 0.83, risk of major CV event: 0.88, risk of myelosuppression, 0.99). Statistical performance was compared across 8 model specifications to determine the specification with the lowest BIC, indicating the best statistical fit. The linear specification with no interaction effect had the lowest (i.e., best) BIC value and was therefore used as the reference model.

## Probabilistic sensitivity analysis: Additional technical details

Uncertainty in the additional benefit offered by ponatinib over imatinib in terms of OS and DOR was represented with 10 000 draws from correlated triangular distributions of additional improvement in OS (∆OS) and additional improvement in DOR (∆DOR). First, draws were taken from a standard multivariate normal distribution with null mean and unit variance. Correlation was between ∆OS and ∆DOR and was introduced with covariance = 0.8. Second, these draws from standard multivariate normal distributions were converted into draws from standard uniform distributions by applying the normal cumulative distribution function. Third, draws from the correlated triangular distributions were obtained by applying the inverse cumulative distribution function of the triangular distributions (mode = 15, 30, or 45, depending on the scenario; minimum = 0 and maximum = 60) to the standard uniform draws.

The use of triangular distributions was guided by 2 constraints. First, we needed to specify one-sided distributions, thus excluding the normal distribution, because the target treatment could not have worse OS or DOR than its competitor. Second, the range of levels for OS included within the DCE was 30 to 90 months, implying that the maximum possible difference between the 2 treatments was 60 months (i.e., OS of 90 months vs. OS of 30 months). Therefore, we needed to use a right-bounded distribution, which thereby excluded the lognormal distribution. Given that the uniform distribution is weakly informative in the context of probability sensitivity analysis because every value within the specified range is equally likely, we selected the triangular distribution. This also made it possible to investigate the impact of the most likely value (i.e., the mode) on the results. The minimum and maximum difference values reflected the minimum and maximum possible differences in OS and DOR included in the DCE (i.e., OS range of 30–90 months; DOR range of 15–75 months).

The “small gain” peaking at 15 months reflected the first quarter of the 0 to 60 month range of differences, the “medium gain” peaking at 30 months reflected the midpoint of the range, and the “large gain” peaking at 45 months reflected the third quarter of the range.

## Table S1. Attribute preferences calculated in the DCE case application [1]

| **Attributes ^a^** | **Levels ^b^** | **MLE (SE)** | **95% CI** |
| --- | --- | --- | --- |
| Alternative specific constant ^c^ | Option A | –0.0350 (0.0443) | [–0.1219; 0.0519] |
|  | Option B | Reference | – |
| Overall survival | 1-month increase | 0.0317 (0.0053)*** | [0.0213; 0.0420] |
| Duration of remission | 1-month increase | 0.0174 (0.0030)*** | [0.0115; 0.0233] |
| Risk of major cardiovascular event | 1% risk decrease | 0.0109 (0.0013)*** | [0.0084; 0.0134] |
| Risk of myelosuppression | 1% risk decrease | 0.0090 (0.0007)*** | [0.0076; 0.0103] |

*** P-value < 0.1%, ** P-value < 1%, * P-value < 5%.

Abbreviations: CI, confidence interval; DCE, discrete choice experiment; MLE, maximum likelihood estimate; MNL, multinomial logit; SE, standard error.

^a^ Attribute definitions for overall survival, duration of remission, major cardiovascular event risk, and myelosuppression are provided in Table 1.

^b^ Attribute levels for overall survival, duration of remission, major cardiovascular event risk, and myelosuppression were linearly coded. Linear encoding assumes linearity in risk preferences, such that a one-unit change in the attribute has a constant effect on participants’ choices and does not depend on the absolute value of the attribute level. Linearity of the 4 attribute preferences was verified by fitting a linear regression; linearity was accepted because each of the regression coefficients was > 0.7 (0.82, 0.83, 0.88, and 0.99, respectively). Statistical performance was compared across eight model specifications to determine the specification with the lowest Bayesian information criterion, indicating the best statistical fit. The linear specification with no interaction effect had the lowest (i.e., best) Bayesian information criterion value and was therefore used as the reference model.

^c^ Preference for the hypothetical treatment alternative labeled “Chemotherapy + TKI A” vs. the hypothetical treatment alternative labeled “Chemotherapy + TKI B”.

## Table S2. Significant preference heterogeneity by personal characteristic and attribute

| **Characteristic (categories)** | **D ^a^** | **Cardiovascular risk** | **Duration of remission** | **Myelosuppression risk** | **Overall survival** |
| --- | --- | --- | --- | --- | --- |
| Number of past Ph+ ALL treatments (0–1; 2–3; more than 3) | 102.15*** | Yes | No | Yes | Yes |
| Remission status: In remission (yes; no) | 51.63*** | Yes | No | Yes | Yes |
| Physical limitations (none; mild; severe) | 49.92*** | No | Yes | Yes | Yes |
| Household income (prefer not to say; below $50k; $50 to $90k; above $90k) | 44.24*** | No | No | Yes | Yes |
| Ph+ ALL-related ER visits (none; ≥ 1) | 42.69*** | No | Yes | No | Yes |
| Number of relapses (none; ≥ 1) | 37.78*** | Yes | No | Yes | Yes |
| Remission status: Not achieved remission (no; yes) | 37.26*** | No | No | Yes | Yes |
| Age (< 40 years, 40–59 years, > 59 years) | 35.84*** | No | No | Yes | Yes |
| Public insurance (yes; no) | 28.26*** | No | No | Yes | Yes |
| Education (less than university, university level) | 25.61*** | No | No | Yes | Yes |
| Private insurance | 25.06*** | No | No | Yes | No |
| Sex (male; female) | 23.61*** | No | No | Yes | Yes |
| Remission status: Just started treatment (yes; no) | 22.97*** | Yes | No | No | Yes |
| Ph+ ALL diagnosis (< 1 year ago; > 1 year ago) | 12.38* | Yes | No | No | Yes |
| Number of current Ph+ ALL treatment (0–1; > 1) | 10.49 | No | Yes | No | No |
| Health literacy (inadequate; adequate) | 9.25 | No | No | No | No |
| Look after children or elderly relatives (living with children or parents; not living with children or parents) | 3.48 | No | No | No | No |
|  | | | | | |

Abbreviations: ER, emergency room; Ph+ ALL, Philadelphia chromosome-positive acute lymphoblastic leukemia.

Note: Yes indicates that preferences were significantly different (P-value < 5%) between categories of the given personal characteristic.

^a^ Deviance from log-likelihood ratio test. *** P-value < 0.1%, ** P-value < 1%, * P-value < 5%.

# References

1. Ashaye A, Thomas C, Dalal M, et al. Patient preferences for frontline therapies for Philadelphia chromosome-positive acute lymphoblastic leukemia: a discrete choice experiment. *Future Oncology*. 2022;18(17):2075-85. <https://doi.org/10.2217/fon-2022-0082>

2. Mattmann M, Logar I, Brouwer R. Choice certainty, consistency, and monotonicity in discrete choice experiments. *Journal of Environmental Economics and Policy*. 2019;8(2):109-27. <https://doi.org/10.1080/21606544.2018.1515118>

3. Segovia MS, Palma MA. Testing the consistency of preferences in discrete choice experiments: an eye tracking study. *European Review of Agricultural Economics*. 2020;48(3):624-64. <https://doi.org/10.1093/erae/jbaa024>

4. Laba T-L, Brien J-a, Jan S. Understanding rational non-adherence to medications. A discrete choice experiment in a community sample in Australia. *BMC Family Practice*. 2012;13(1):61. <https://doi.org/10.1186/1471-2296-13-61>
